# Dance movement therapy for neurodegenerative diseases: A systematic review

**DOI:** 10.3389/fnagi.2022.975711

**Published:** 2022-08-08

**Authors:** Cheng-Cheng Wu, Huan-Yu Xiong, Jie-Jiao Zheng, Xue-Qiang Wang

**Affiliations:** ^1^Department of Sport Rehabilitation, Shanghai University of Sport, Shanghai, China; ^2^Huadong Hospital, Shanghai, China; ^3^Department of Rehabilitation Medicine, Shanghai Shangti Orthopaedic Hospital, Shanghai, China

**Keywords:** dance movement, aging, neurodegenerative disease, Parkinson's disease, mild cognitive impairment, Alzheimer's disease, neurorehabilitation

## Abstract

**Background:**

The proportion of the world's elderly population continues to rise, and the treatment and improvement of neurodegenerative diseases have become issue of public health importance as people live longer and many countries have aging populations. This systematic review aims to discuss the effects of dance movement therapy (DMT) on motor function, cognitive deficit, mood, and quality of life in people with neurodegenerative diseases, such as Parkinson's disease (PD), mild cognitive impairment (MCI), Alzheimer's disease (AD).

**Methods:**

Two reviewers independently conducted systematic search on the Cochrane library, PubMed database, Web of Science Core Collection database, and Physiotherapy Evidence database until February 1, 2022. Only systematic analyses and randomized controlled trials were included and further analyzed.

**Results:**

Thirty-three studies on PD, 16 studies on MCI, 4 studies on AD were obtained. This systematic review found that DMT substantially improved the global cognitive function, memory, and executive function on the population with MCI. Compared with the non-dance group, DMT remarkably improved general disease condition, balance, and gait for individuals with PD. The evidence of the efficacy of DMT on AD is insufficient, and further research is needed.

**Conclusion:**

DMT can effectively improve the motor function and cognitive deficits in neurodegenerative diseases. Positive effects of DMT on the mood and quality of life in ND patients are controversial and require further evidence. Future research on the effects of DMT on AD requires scientific design, large sample size, long-term comprehensive intervention, and clear reporting standards.

**Systematic review registration:**

www.osf.io/wktez, identifier: 10.17605/OSF.IO/UYBKT.

## Introduction

Eurostat forecasts show that the proportion of the population over 60 years old will reach 35%, and the number of people aged 65 and over with Alzheimer's disease (AD) in the United States may increase to 13.8 million by the middle of this century (2020 Alzheimer's disease facts and figures, 2020). Aging is the primary risk factor for most neurodegenerative diseases (NDs), which include Parkinson's disease (PD), mild cognitive impairment (MCI) and AD. One in 10 individuals aged ≥65 suffering from NDs will increase with the proportion of the elderly population, which will cause a great burden on individuals, families, and society (Hou et al., 2019). Therefore, the treatment and improvement of NDs with aging as a major risk factor have become the issue of public health that needs to be solved urgently in the medical and health field. Previous studies suggested that the functional recovery of patients with NDs depends on the ability of spare neurons to rebuild and reshape the damaged network to compensate for the lost function by growing neurites and forming new synapses (Reetz et al., 2010; Tomassini et al., 2012; Nudo, 2013). This traditional strategy of function is considered to achieve rehabilitation through targeted training of weakened functions (Herholz et al., 2013; Zeiler and Krakauer, 2013; Agosta et al., 2017). Another method is to increase the overall level of brain activity through sensory and cognitive stimulation (Baroncelli et al., 2010).

Dance is a rhythmic movement that has been choreographed or improvised in advance and usually performed with music. The American Dance Therapy Association (ADTA) defines dance movement therapy (DMT) as “the psychotherapeutic use of movement as a process that promotes the emotional, social, cognitive, and physical integration of the individual.” Formal DMT can be defined as active interventions (such as performing tango, waltz, dances) or receptive interventions (such as watching stage plays) by qualified dance therapists (groups). The meta-analysis results of Fong Yan et al. (2018) confirmed that the therapeutic effect of performing any type of DMT is better than other types of structured exercise in improving a series of health outcomes, such as body composition and musculoskeletal function (Fong Yan et al., 2018). Many studies have reported the positive effects of DMT in various conditions, such as cancer (Aktas and Ogce, 2005; Bradt et al., 2015), brain health (Rios Romenets et al., 2015; Karkou and Meekums, 2017; Poier et al., 2019; Ruiz-Muelle and López-Rodríguez, 2019), cardiovascular disease (Conceição et al., 2016; Fong Yan et al., 2018; Gronek et al., 2021), and fall (Fernández-Argüelles et al., 2015; Veronese et al., 2017). Studies examining the effects of DMT have mostly focused on the population with NDs, particularly PD. Various neuroimaging studies of dance observation showed that DMT can improve the neuronal connectivity in specific brain regions of older participants' brain, promote neuroplasticity, and induce changes in gray and white matter in multiple brain regions, especially the frontotemporal region. Previous studies suggested DMT as a rehabilitation tool of disease management and clinical improvement for patients with AD (Mabire et al., 2019; Ruiz-Muelle and López-Rodríguez, 2019), MCI (Liu et al., 2021; Wang et al., 2021; Wu et al., 2021), and PD (Barnish and Barran, 2020; Berti et al., 2020; Carapellotti et al., 2020; de Almeida et al., 2020; Emmanouilidis et al., 2021; Ismail et al., 2021). The pooled results of the latest meta-analysis of the efficacy of DMT in PD patients showed that DMT can improve motor function and reduce the burden on caregivers (Emmanouilidis et al., 2021; Ismail et al., 2021). The meta-analysis findings of randomized controlled trials (RCTs) and non-RCTs reported the positive effects of DMT in improving the motor, cognitive, and psychological functions in patients with AD (Ruiz-Muelle and López-Rodríguez, 2019). Among the studies on the dance treatment of MCI, many systematic reviews and meta-analyses confirmed that DMT can improves memory and other cognitive function, psychological function, and quality of life (Zhu et al., 2020; Liu et al., 2021; Wu et al., 2021).

At present, there are various types of dance-movement therapy and widely accepted by groups of all ages. For example, the Videogame-Based Dance Exercise Program attracts many young people, and Chinese-style square dance is also recognized by middle-aged and elderly groups. In the foreseeable future, there will be a variety of ways for people to access dance movement therapy, no longer limited to one-on-one or one-to-many treatment modes between dance therapists and patients. This also provides one of the treatment options for at-home rehabilitation for the elderly population who have limited cognition, activity and social skills due to aging. However, extant studies only summarized some aspects of the effectiveness of DMT for a certain NDs. A comprehensive overview and interpretation of the benefits and potential mechanisms of DMT in the rehabilitation of major neurodegenerative diseases with aging as a major risk factor are needed. We conducted an integrated review of systematic analyses and RCTs of DMT in patients with PD, MCI, AD and discussed the underlying mechanisms of the efficacy of DMT.

## Materials and methods

This protocol of this systematic review has been registered in the Open Science Framework (OSF) systematic review database, international prospective register for systematic review under the registration 10.17605/OSF.IO/UYBKT.

### Search strategy and selection criteria

We searched the literature *via* the PubMed database, Web of Science Collection database, Cochrane library, and Physiotherapy Evidence Database (PEDro). No limit was set on data search. The key words of search strategies were as follows: “dance,” “Alzheimer's disease,” “cognitive impairment,” “Parkinson's disease,” (Supplementary material).

Studies were chosen on the basis of four criteria: 1. Studies must be written in English language; 2. The types of studies were limited to systematic analysis and RCT; 3. Only dance was used as a patient-specific intervention; 4. Studies should include patients with MCI, PD and AD. 5. Studies must be published before February 1, 2022. All articles were screened by title and abstract. When the article may be a systematic analysis or RCT, the full text was queried for quality assessment, and a list analysis was performed.

### Data collection, extraction, and quality assessment

Two reviewers independently conducted the literature search, data extraction, and quality assessment. For each eligible systematic analysis or RCT, we extracted and developed the characteristics for the included study, the size of participants and conditions, professional dance therapists involved, blind method setting, primary outcome, main results, and overall duration of intervention (Tables 1–4). Periodic study group meetings were held to review the process, and disagreements were resolved by consensus or referral to a third reviewer.

**Table 1:** Randomized controlled trials assessing various dance-based movement interventions in patients with mild cognitive impairment or Alzheimer's disease.

**Experimental group vs. control group; (Participants: n)**	**Primary outcome**	**Overall duration of intervention**	**Main results**
**Mild cognitive impairment**			
Aerobic dance routine vs. usual care; (60) (Zhu et al., 2018)	(1) MoCA; (2) WMS-R; (3) DST; (4) SDMT; (5) TMT; (6) FAQ; (7) SF-36; (8) GDS-15; (9) ERP; (10) Adverse events;	21 h in 12 weeks; No reported.	(2) DG vs. CG: mean change = 4.6; 95% CI = 2.2 to 7.0; *p =* 0.001 (at 3 months); (1); (3); (4); (5); (6); (7); (8); (9); (10) NS (between-group difference);
Aerobic dance vs. health education; (68) (Zhu et al., 2022)	(1) WMS-RLM; (2) Unilateral hippocampal volume;	21 h in 12 weeks; 88.6%.	(1) DG vs. CG: β = 0.326, 95%CI = 1.005 to 6.773, *p =* 0.009; (2) DG vs. CG: β = 0.379, 95% CI = 0.117 to 0.488, *p =* 0.002 (right); β = 0.344, 95% CI = 0.082, 0.446, *p =* 0.005 (total);
Dance vs. a life as usual; (62) (Rektorova et al., 2020)	(1) MoCA; (2) TFT; (3) Logical memory; (4) FPT; (5) ToH; (6) JLO; (7) MAST; (8) BADLS;	60 h in 24 weeks; No reported.	(1); (2); (3) DG vs. CG: NS (between-group difference); (4) DG vs. CG: F = 4.07, *p =* 0.05; (5); (6); (7); (8); (9) DG vs. CG: NS (between-group difference);
Specially designed moderate-intensity aerobic dance vs. usual care; (32) (Qi et al., 2019)	(1) MMSE; (2) MoCA; (3) WMS-R LM; (4) DST-F; DST-B; (5) SDMT; (6) BBS;	21 h in 12 weeks; No reported.	(1); (2) DG vs. CG: NS (between-group difference); (3) *p < * 0.05; (4); (5); (6) DG vs. CG: NS (between-group difference);
Participants familiarized dance vs. usual care; (129) (Lazarou et al., 2017)	(1) MMSE; (2) MoCA; (3) FUCAS; (4) TEA-4; (5) RAVLT; (6) RBMT; (7) FAS; (8) ROCFT; (9) GDS; (10) NPI;	80 h in 40 weeks; No reported.	(1) DG vs. CG: *p < * 0.001; (2) DG vs. CG: *p =* 0.03; (3) NS (between-group difference); (4) DG vs. CG: *p =* 0.002; (5) DG vs. CG: *p =* 0.003; (6) DG vs. CG: RBMT-1, *p =* 0.004; RBMT-2, *p =* 0.001; (7) DG vs. CG: *p =* 0.005; (8) DG vs. CG: *p < * 0.001; (9) DG vs. CG: *p =* 0.022; (10) DG vs. CG: *p =* 0.02;
Dance vs. life-as-usual; (99) (Kropacova et al., 2019)	(1) TCF 1; (2) TCF 2; (3) WMS III: LogPam; (4) LogPam2; (5) DigitSpan; (6) WAIS III: Symbols; (7) ToH 3; (8) ToH 4; (9) FPT; (10) JLO;	72 h in 24 weeks; 78.1%.	(1) (2); (3); (4); (5); (6); (7); (8); (10): DG vs. CG: NS (between-group difference); (9) DG vs. CG: *p =* 0.008;
Dance, music, and health education; (201) (Doi et al., 2017)	(1) Story memory; (2) Word memory;	40 h in 40 weeks; 93%.	(1) DG vs. CG: *p =* 0.011; (2) NS (between-group difference);
Dance vs. music; (100) (Cross et al., 2012)	(1) BDI; (2) RMT-F;	0.5 h; No reported.	(1) DG vs. MG: *p < * 0.001 (at after-intervention 3 days); *p =* 0.008 (at after-intervention 10 days); (2) DG vs. MG: *p =* 0.002 (at after-intervention 3 days);
Chinese square dance vs. usual care; (109) (Chang et al., 2021)	(1) MoCA; (2) SF-12; (3) GDS-15; (4) BBS;	27h in 18 weeks; 19.9%.	(1) DG vs. CG: NS (group*time effect, at 9 weeks); *p =* 0.004 (group*time effect, at 18 weeks); (2) DG vs. CG: MCS, *p =* 0.004 (group*time effect, at 9 weeks); *p =* 0.001 (group*time effect, at 18 weeks); PCS, NS (group*time effect, at 9 weeks and 18 weeks); (3) DG vs. CG: NS (group*time effect, at 9 weeks); *p =* 0.009 (group*time effect, at 18 weeks); (4) DG vs. CG: NS (group*time effect, at 9 weeks); NS (group*time effect, at 18 weeks);
Fitness-dance vs. life as usual; (12) (Ammar et al., 2021)	(1) CERAD-Plus; (2) PAR-Q; (3) Physical performance and heart rate;	24 h in 8 weeks; No reported.	(1); (2); (3) DG vs. CG: NS (between-group difference);
BAILAMOS vs. waitlist control; (21) (Aguiñaga and Marquez, 2017)	Focus groups;	32h in 16 weeks. 100%	The focus group data revealed that participants were energized by the dance program, and they enjoyed learning new dance styles and techniques.
**Alzheimer's disease**			
Dance groups vs. music appreciation and socialization groups; (18) (Low et al., 2016)	(1) The number of falling; (2) Neuropsychological tests; (3) Standing balance, walking speed and sit to stand speed: (4) Global functioning	36h in 12 weeks; 67%.	There were no significant differences between the groups.

MCI, Mild cognitive impairment; MoCA, The Montreal Cognitive Assessment; WMS-R, Wechsler Memory Scale-revised logical memory test; SDMT, Symbol Digit Modalities Test; TMT, Trail Making Test; DST, Forward and backward Digit Span Task; FAQ, Functional Activities Questionnaire; SF-36, The 36-item Short Form Health Survey; GDS-15, The 15-item Geriatric Depression Scale (GDS-15); ERP, event-related potential; DG, dance group; CG, control group; NS, none significant; TFT, Taylor Figure Test; FPT, Five-Point Test; ToH, Tower of Hanoi; JLO, Judgment of Line Orientation; MAST, Mississippi Aphasia Screening Test; BADLS, Bristol Activities of Daily Living Scale; WMS-R LM, Wechsler Memory Scale-Revised Logical Memory; DST-forward, Digit Span Test-F; DST-backward, DST-B; SDMT, Symbol Digit Modalities Test; BBS, Berg Balance Scale; FUCAS, Functional and Cognitive Assessment Test; TEA-4, Test of Everyday Attention; RAVLT, Rey Osterrieth Complex Figure Test copy and delay recall; RBMT, Rivermead Behavioral Memory Test; FAS, The Verbal Fluency F-A-S Test; ROCFT, Rey Osterrieth Complex Figure Test; GDS, Global Deterioration Scale; NPI, Neuropsychiatric Inventory; BDI, Beck Depression Inventory; RMT-F, Recognition Memory Test–Faces; MG, music group; SF-12, The Short-Form 12 Health Survey; GDS-15, The Geriatric Depression Scale; CERAD-Plus, Consortium to Establish a Registry for Alzheimer's Disease.

**Table 2:** Randomized controlled trials assessing various dance-based movement interventions in patients with Parkinson's disease.

**Experimental group vs. control group**	**Primary outcome**	**Overall duration of intervention; participant rate (%)**	**Main results**
Irish set dancing classes vs. physiotherapy; (24) (Volpe et al., 2013)	(1) MDS-UPDRS-III; (2) TUG; (3) BBS; (4) FOG-Q;	36h (mean: 32.745h) in 24 weeks; 90.9%.	(1) DG vs. CG: F = 6.35, *p =* 0.019; (2) DG vs. CG: F = 8.938, *p =* 0.007; (3) DG vs. CG: NS (between-group difference, F = 4.254, *p =* 0.051); (4) DG vs. CG: t = 16.296, *p < * 0.001;
Irish set dancing classes vs. exercises or usual care; (41) (Shanahan et al., 2017)	(1) UPDRS-III; (2) 6 MWT; (3) Mini-BESTest; (4) PDQ-39;	25h in 10 weeks; 93.5%.	(1) Postintervention, the dance group had greater nonsignificant gains in quality of life than the usual care group; (2) (3) (4) (5) DG vs. CG: NS (between-group difference)
Sardinian folk dance vs. usual care; (20) (Solla et al., 2019)	(1) UPDRS-III; (2) 6MWT; (3) FTSST; (4) TUG test; (5) BBS; (6) MIMUs; (6) MoCA; (7) BDI-II; (8) SAS;	36h in 12 weeks; 92.9%.	(1) DG vs. CG: F = 22.191, *p < * 0.001; (2) DG vs. CG: F = 41.124, *p < * 0.001; (3) DG vs. CG: F = 95.685; *p < * 0.001; (4) DG vs. CG: F = 26.014; *p < * 0.001; (5) DG vs. CG: F = 49.834; *p < * 0.001; (5) Stride length: DG vs. CG, F = 5.608; *p =* 0.03; Walking speed: DG vs. CG, F = 4.524; *p =* 0.049; Walking cadence: DG vs. CG, F = 4.572; *p =* 0.048; GFI revealed: DG vs. CG, F = 10.797; *p =* 0.005; (6) DG vs. CG: F = 7.913; *p =* 0.012; (7) DG vs. CG: F = 47.957; *p < * 0.001; (8) DG vs. CG: F = 7.106; *p =* 0.016
Tango, parted vs. usual care; (33) (Rios Romenets et al., 2015)	(1) the MDS-UPRDS-3; (2) Mini-BESTest; (3) TUG; (4) Dual-task TUG; (5) Fall; (6) FOG-Q; (7) FSS; (8) Upper extremity function; (9) MoCA; (10) BDI; (11) AS;	24h in 12 weeks; 61%.	(1) (5) (6) (7) (10): DG vs. CG: NS (between-group difference); (2) DG vs. CG: *p =* 0.032; (3) DG vs. CG: *p =* 0.042; (4) DG vs. CG: *p =* 0.012; (8) DG vs. CG: *p =* 0.038 (After multivariate adjustment for baseline average time on exercise/dance); (9) DG vs. CG: *p =* 0.01 (after exclusion of the 9 protocol violations);
Tango single vs. usual care; (13) (Michels et al., 2018)	(1) MDS-UPRDS-3; (2) Hoehn and Yahr scale;	10h in 10 weeks; Not reported.	The study was not powered to assess whether any of these differences were statistically significant.
Tango vs. Tai Chi; (29) (Poier et al., 2019)	(1) the MDS-UPRDS-3; (2) The MoCA; (3) PDQ-39; (4) BMLSS; (5) BDI; (6) FSS; (7) VAFS;	10h in 10 weeks; Not reported.	(1) (2) (3) (4) (5) (6) (7): NS (between-group difference)
Group/Partnered vs. group structured strength/flexibility exercise; (19) (Hackney et al., 2007)	(1). The MDS-UPRDS; (2) BBS; (3) TUG and Dual-Task TUG; (5) Freezing of Gait; (6) Walking and Dual-Task Walking;	21h in 13 weeks; 100%.	(1) (2) (3) (4) (5) (6): NS (between-group difference)
Partnered vs. Nonpartnered tango; (39) (Hackney and Earhart, 2010)	(1) The MDS-UPRDS-3;	20h in 10 weeks; 80%.	No group comparisons were made in this RCT.
Tango vs. waltz/foxtrot or no intervention (control) groups; (58) (Hackney and Earhart, 2009)	(1) The MDS-UPRDS-3; (2) BBS; (3) TUG; (4) 6 MWT; (5) FOG; (6) TUG; (7) Gait speed, stride length, and single support time;	20h in 13 weeks; Not reported.	(1) (3) (5) (6) Tang vs. CG: NS (between-group difference); Waltz/foxtrot vs. CG: NS (between-group difference); (2) Tang vs. CG: *p < * 0.05; Waltz/foxtrot vs. CG: *p < * 0.05; (4) Tang vs. CG: *p < * 0.05; Waltz/foxtrot vs. CG: *p < * 0.05; (7) Tang vs. CG: backward stride length, *p < * 0.05; Waltz/foxtrot vs. CG: backward stride length, *p < * 0.05;
Partnered community-based tango vs. no intervention control group; (52) (Foster et al., 2013)	(1) UPRDS-1 and 3; (2) BDI; (3) ACS;	96h in 48 weeks; Not reported. 90%	These patterns were similar in the separate activity domains. The tango group gained a significant number of new social activities (*p =* 0.003), but the control group did not (*p =* 0.71).
Partnered community-based tango vs. no intervention control group; (62) (Duncan and Earhart, 2012)	(1) MDS-UPRDS-3; (2) Mini-BESTest; (3) FOG-Q; (4) 6 MWT; (5) Walking velocity during comfortable forward, fast as possible forward, dual task, and backward walking; (6) 9HPT;	96h in 48 weeks; 78.5 ± 3%.	(1) DG vs. CG: total scores, F = 9.82, *p < * 0.001; tremor scores, NS (between-group difference); rigidity, F = 11.72, *p < * 0.001 (time*group effect, at 6 and 12 months); Bradykinesia, F = 8.35, *p < * 0.001 (time*group effect, at 6 and 12 months); (2) DG vs. CG: F = 11.73, *p < * 0.001 (time*group effect, at 3, 6 and 12 months); (3) DG vs. CG: NS (between-group difference); (4) DG vs. CG: distance of walking, *p < * 0.05; (5) DG vs. CG: forward walking velocity, *p < * 0.05 (between-group difference, at 6 and 12 months); dual-task walking, F = 3.57, *p =* 0.02; (6) DG vs. CG: F = 3.83, *p =* 0.01;
Partnered community-based tango vs. no prescribed exercise control group; (10) (Duncan and Earhart, 2014)	(1) MDS-UPRDS-1, 2, and 3; (2) Mini-BESTest; (3) gait velocity (forward and backward); (4) TUG and dual-task TUG; (5) 6MWT; (6) FOG-Q;	192 h in 96 weeks; Not reported.	(1) DG vs. CG: MDS-UPRDS-3, F= 17.59; *p < * 0.001 (time*group effect, at 12 and 24 months); UPRDS-2, F = 3.53; *p =* 0.05 (time*group effect, at 12 and 24 months); UPRDS-1, F = 5.10; *p =* 0.02 (time*group effect, at 12 and 24 months); (2) DG vs. CG: F = 11.33; *p < * 0.001 (time*group effect, at 12 and 24 months); (3) (6) DG vs. CG: NS (between-group difference); (4) DG vs. CG: TUG, NS (between-group difference); Dual-Task TUG, F = 3.7; *p =* 0.048 (time*group effect, at 12 and 24 months); (5) DG vs. CG: F = 5.67; *p =* 0.013 (time*group effect, at 12 and 24 months);
Turo (mixed Qigong dance); (20) (Lee et al., 2018)	(1) UPDRS; (2) ADL; (3) PDQ-39; (3) BDI-21; (4) BBS;	8 weeks; Not reported.	(1) DG vs. CG: *p =* 0.001; (2) DG vs. CG: *p =* 0.002; (3) DG vs. CG: *p =* 0.049; (4) DG vs. CG: NS (between-group difference, *p =* 0.051);
Double ballroom and Latin American dance vs. usual care; (27) (Hulbert et al., 2017)	Twelve, 180° on-the-spot turns	20h in 10 weeks; Not reported.	Significant 4-way interactions between the groups, over time and turn style, with longer latency of the head (*p =* 0.008) and greater rotation in the pelvis (*p =* 0.036), alongside a trend of slower movement of the first (*p =* 0.063) and second (*p =* 0.081) foot in controls were shown, with minimal change in dancers.
Incorporated strategies-based dance vs. PD exercise; (46) (Hashimoto et al., 2015)	(1) UPDRS; (2) TUG; (3) BBS; (4) FAB; (5) MRT; (6) AS; (7) SDS;	12h in 12 weeks; Not reported.	(1) DG vs. CG: *p < * 0.001; (2) DG vs. CG: TUG time, *p =* 0.006, TUG step number, *p =* 0.005; (3) DG vs. CG: *p =* 0.001; (4) DG vs. CG: *p =* 0.001; (5) DG vs. CG: MRT response time, *p < * 0.001; (6) DG vs. CG: *p < * 0.001; (7) DG vs. CG: *p =* 0.006;
Dance-physiotherapy combined intervention vs. conventional physiotherapy; (38) (Frisaldi et al., 2021)	(1) MDS-UPDRS-III; (2) 6 MWT; (3) TUG; (4) Mini BESTest; (5) New FOG-Q; (6) MoCA;(7) TUG-DTT; (8) PDQ-39; (9) BDI;	15h in 5 weeks; 100%	(1) DG vs. CG: MD = −2.72, 95% CI = −5.28 to −0.16, *p =* 0.038; (2) (3) (4) DG vs. CG: NS (between-group difference); (5) Significant improvements were only found in the control group; (6) Significant improvement was only found in the dance group (*p =* 0.03); (7) Significant improvement was only found in the dance group (*p =* 0.02); (8) DG vs. CG: NS (between-group difference); (9) Significant improvement was only found in the control group;
Binary vs. quaternary dance rhythm; (31) (Moratelli et al., 2021)	(1) Hoehn and Yahr scale; (2) UPDRS-1, and 2; (3) MMSE; (4) MoCA; (5) PDQ-39; (6) mental activity;	18h in 12 weeks; 84.3%	Both intervention groups improved cognition (MoCA: *p < * 0.001, d = 0.05), mental activity (UPDRS-1: *p < * 0.001). UPDRS-1 items, the QG was highlighted in intellectual impairment (*p* = 0.005) and motivation (*p =* 0.021).
Virtual reality dance exercise vs. usual care; (20) (Lee et al., 2015)	(1) BBS; (2) MBI; (3) BDI;	15h in 6 weeks; Not reported.	(1) DG vs. CG: *p < * 0.05; (2) DG vs. CG: *p < * 0.05; (3) DG vs. CG: *p < * 0.05;

PD, Parkinson's disease; UPDRS-III, Unified Parkinson's Disease Rating Scale Part-III; TUG, Timed Up-and-Go; BBS, Berg Balance Scale; FOG, Freezing of Go Questionnair; MD, Mean Difference; 6 MWT, 6-minute walk test; Mini-BESTest, Mini-Balance Evaluation Systems Test; PDQ-39, Parkinson's disease questionnaire-39; FTSST, the Five Times Sit-to-Stand Test; SRT, Sit-and-Reach Test; BST, Back Scratch Test; MIMUs, MagnetoInertial Measurement Units; PFS-16, Fatigue Scale; BDI-II, The Beck Depression Inventory; SAS, Starkstein Apathy Scale; FSS, Fatigue Severity Scale; AS, Apathy Scale; VAFS, Visual Analog Fatigue Scale; BMLSS, Brief Multidimensional Life Satisfaction Scale; ACS, the Activity Card Sort; 9HPT, the Nine-Hole Peg Test; MRT, Mental Rotation Task; SDS, Self-rating Depression Scale; MBI, the Modified Barthel Index; STAIL, State-Trait Anxiety Inventory; FES-1, Falls Efficacy ScaleInternational; TRAIL, Trail Making Test; MCS, the mental component summary; PCS, the physical component summary; PAR-Q, The Modified German version of the Physical Activity Readiness Questionnaire; yr, year.

**Table 3:** Systemic reviews assessing various dance-based movement interventions in patients with mild cognitive impairment or Alzheimer's disease.

**Author (Year; Studies; Participants)**	**Primary outcome**	**Main results**
**Mile cognition impairment**		
Zhu et al. (2020; 5 RCTs; 644; 8)	(1) FAS;(2) TMT; (3) Immediate recall ability; (4) Delayed recall;	Compared to control: (1) MD = 1.73, 95%CI = 0.58 to 2.88, *p =* 0.003; (2) TMT-A, MD = −2.37, 95%CI = −4.16 to 0.58; *p =* 0.010; TMT-B, MD = −16.07, 95%CI = −30.03 to 0.01, *p =* 0.020; (3) SMD = 0.24, 95%CI = 0.01 to 0.46, *p =* 0.04; (4) SMD = 0.46, 95%CI = 0.30 to 0.62, *p < * 0.001;
Wu et al. (2021; 8 RCTs; 548; 11)	(1) Global cognition; (2) Memory; (3) Visuospatial function; (4) Language; (5) Motor function;	Compared to control: (1) SMD = 0.54, Z = 3.55, *p < * 0.001; (2) SMD = 0.33, Z = 3.97, *p < * 0.001; (3) SMD = 0.42, Z = 2.41, *p =* 0.02; (4) SMD = 0.39, Z = 2.69, *p =* 0.007; (5) SMD = 0.93, Z = 5.04, *p < * 0.001;
Wang et al. (2021; 5; 579; 9)	(1) Depression; (2) Anxiety;	Compared to control: (1) SMD = −0.42, 95%CI = −0.60 to −0.23, *p < * 0.05; (2) NS (between-group difference)
Liu et al. (2021; 12 RCTs; 896; 9)	(1) Global cognition; (2) Executive function; (3) Immediate Recall; (4) Delayed Recall; (5) Language;	Compared to control: (1) SMD = 0.73, 95%CI = 0.47 to 0.99, *p < * 0.001; (2) NS (MD = −3.16, 95%CI = −7.16 to −0.85, *p =* 0.12); (3) SMD = 0.54, 95%CI = 0.30 to 0.78, *p < * 0.0001; (4) SMD = 0.56, 95%CI = 0.26 to 0.86, *p =* 0.0002; (5) *p < * 0.05;
Chan et al. (2020; 5 RCTs; 358; 9)	(1) Global cognition; (2) Attention; (3) Immediate Recall; (4) Delayed Recall; (5) Visuospatial ability;	Compared to control: (1) SMD = 0.48, 95%CI = 0.21 to 0.74, *p < * 0.05; (2) SMD = 0.33, 95%CI = 0.12 to 0.54, *p < * 0.05; (3) SMD = 0.54, 95%CI = 0.38 to 0.71, *p < * 0.05; (4) SMD = 0.33, 95%CI = 0.01 to 0.64, *p < * 0.05; (5) SMD = 0.16, 95%CI = 0.01 to 0.32, *p < * 0.05;
**Alzheimer's disease**		
Karkou and Meekums (2017; 0; 0; 8)	(1) Challenging behaviors; (2) Cognitive function; (3) Depression; (4) Quality of life;	None of which met the inclusion criteria.
Mabire et al. (2019; 14 studies; 967; 5)	(1) Motor function; (2) Psychological function; (3) Cognitive function; (4) Quality of life; (5) Self-esteem; (6) Social interactions; (7) Behavioral outcomes;	This review found that nine practice recommendations for implementing dance interventions were identified according to primary intentions of the intervention (therapeutic or recreational): indications; contra-indications; participant profile; dosage; session sequencing; setting of intervention; observance/attendance; contributors and facilitators; and assessments.
Ruiz-Muelle and López-Rodríguez (2019; 12 studies; 349; 8)	(1) Physical function; (2) Cognitive function; (3) Psychological function; (4) Quality of life; (5) Burden of Care;	This mini-review confirmed the positive effect of dance therapy on physical and cognitive function, functionality, psychological outcomes, and quality of life in cognitive dysfunction people

RCT, randomized control trial; AD, Alzheimer's disease; Quality of Life; MCI, mild cognition impairment; FAS, The Verbal Fluency F-A-S Test; TMT, Trail Making Test; MD, Mean Difference; CI, Confidence Interval; SMD, Standardized Mean Difference; NS, No significant; High quality: total score ≥7; moderate quality: total score 4–6; low quality: total score ≤3.

**Table 4:** Systemic reviews assessing various dance-based movement interventions in patients with Parkinson's disease.

**Author (Year; Studies; Participants; Quality)**	**Primary outcome**	**Main results**
Zhang Q. et al., (2019; 7 RCTs; 185; 7)	(1) MoCA; (2) FAB; (3) SDS; (4) BDI; (5) AS;	Compared to control: (1) WMD = 2.02, 95%CI:0.65 to 3.38, *p =* 0.004; (2) WMD = 1.17, 95%CI:0.39 to 1.95, *p =* 0.003; (3) (4) (5) NS;
Sharp and Hewitt (2014; 5 RCTs; 143; 10)	(1) UPDRS−3 motor scores; (2) Gait speed; (3) Balance; (4) PDQ−39;	Compared to no treatment: (1) MD = −10.73, 95%CI = −15.01 to −6.16, *p =* 0.004; (2) MD = 0.14m/s, 95%CI = 0.02 to 0.26, *p =* 0.02; (3) MD = 0.72, 95%CI = 0.31 to 1.44, *p < * 0.001; compared with other exercise: MD = 3.98, 95%CI = 1.52 to 6.44, *p =* 0.002; (4) Compared with other exercise: MD = −4.00, 95%CI = −7.13 to −0.87, *p =* 0.01.
Shanahan et al. (2015; 12 studies; 359; 6)	(1) UPDRS−3; (2) BBS; (3) TUG; (4) 6–MWT; (5) PDQ−39; (6) PAS;	In this review, dance was found to be more effective than a control intervention for improving balance, motor impairment. Two 1–hour dance classes per week, for at least 10 weeks, can have positive effects. Greater benefit might also be seen with longer duration interventions
Lötzke et al. (2015; 12 sudies; 433; 6)	(1) UPDRS−3; (2) The Mini–BESTest; (3) BBS; (4) TUG; (5) 6 MWT; (6) FOG–Q;	Compared to control: (1) 95%CI = −1.04 to −0.21, *p < * 0.05; (2) 95%CI = 0.60 to 1.31, *p < * 0.05; (3) 95%CI = 0.01 to 0.90, *p < * 0.05 (4) 95%CI = −0.72 to −0.2, *p < * 0.05; (5) (6) NS, p> 0.05
Kalyani et al. (2019; 12 studies; 589; 10)	(1) UPDRS−3; (2) Gait speed; (3) TUG; (4) FOG–Q; (5) 6 MWT; (6) Dual–task TUG; (7) MoCA	Compared to control: (1) SMD = −1.04, 95%CI = −1.69 to −0.39, *p < * 0.05; (2) NS (SMD = 0.37, 95%CI = −0.13 to 0.86); (3) SMD = −0.54, 95%CI = −0.91 to −0.16, *p < * 0.05; (4) NS (SMD = −0.38, 95%CI = −0.09 to 0.34); (5) SMD = 0.75, 95%CI = 0.15 to 1.35, *p < * 0.05; (6) SMD = −0.85, 95%CI = −1.50 to −0.21, *p < * 0.05; (7) SMD = 0.52, 95%CI= −0.00 to 1.04, *p < * 0.05;
Ismail et al. (2021; 20 RCTs; 723; 6)	(1) MDS–UPDRS−1; (2) MDS–UPDRS−2; (3) MDS–UPDRS−3; (4) MDS–UPDRS−4; (5) The Mini–BEST Test; (6) BBS; (7) FOG–Q; (8) TUG; (10) 6 MWT; (11) BST;	Compared to no treatment: (1) MD= −3.50, 95%CI = −18.68 to 11.67, *p < * 0.05; (2) MD = −2.09, 95%CI=-7.57 to 3.40, *p < * 0.05; (3) MD = −6.91, 95%CI = −9.97 to −3.84, *p < * 0.05 (at 3 months); (4) NS (MD= −0.10, 95%CI= −0.79 to 0.59) (5) MD= 4.47, 95%CI = 2.29 to 6.66, *p < * 0.05; (6) MD = 8.42, 95%CI = 3.68 to 13.17, *p < * 0.05 (at 3 months);; (7) MD = −0.39, 95%CI= −2.99 to 2.24, *p < * 0.05; (8) MD = −1.16, 95%CI = −2.17 to −0.15, *p < * 0.05; (9) MD = 238.80, 95%CI = 157.99 to 319.61, *p < * 0.05; (10) NS (MD = 5.30, 95%CI= −2.94 to 13.54); (11) NS (RR = 0.56, 95%CI = 0.11 to 2.90);
Emmanouilidis et al. (2021; 39 studies; 1198; 7)	(1) Gait; (2) Balance; (3) Movement; (4) Mobility; (5) Movement disorders; (6) Participation;	This review found that there are positive associations between therapeutic dancing and improvements in gait, balance, movement disorders, and disability.
Dos Santos Delabary et al. (2018; 5 RCTs; 159; 8)	(1) UPDRS−3; (2) TUG; (3) 6 MWT; (4) FOG–Q; (5) Velocity of forward and backward walking; (6) PDQ−39;	Compared to control: (1) CI= −13.79 to 2.91, *p =* 0.003; (3) NS (CI= −6.72 to 79.19, *p =* 0.10); (4) NS (CI= −4.95 to 0.29, *p =* 0.08); (5) Forward waling, Cl = 0.33 to 0.20, *p =* 0.15; Backward Walking, CI = −0.09 to 0.24, *p =* 0.38; (6) NS (CI = −8.33 to 4.26, *p =* 0.53); (2) Compared to other exercise: CI = −2.03 to −0.27, *p =* 0.01;
Carapellotti et al. (2020; 16 RCTs; 636; 9)	(1) Motor outcome; (2) Cognitive function; (3) Mental health related outcomes; (4) Quality of life;	The reviewed evidence demonstrated that dance can improve motor impairments, specifically balance and motor symptom severity in individuals with mild to moderate PD, and that more research is needed to determine its effects on non–motor symptoms and quality of life.
Berti et al. (2020; 21 studies; 383; 7)	The Template for Intervention Description and Replication guidelines and checklist were used to assess quality and quantity of the content of Argentine tango interventions' description.	This review found that the included RCT interventions were well described, such as details of intervention procedures and doses. In addition, participants in the dance intervention showed strong adaptability and compliance.
Hidalgo-Agudo et al. (2020; 11 RCTs; 982; 8)	(1) UPDRS–III; (2) TUG; (3) BBS; (4) ABC; (5) FES; (6) PDQ−39;	Compared to no treatment: (1) NS (*p =* 0.14); (2) MD = −1.16, 95%CI = −2.30 to −0.03, *p =* 0.04; (3) MD = 4.05, 95%CI = 1.34 to 6.75, *p =* 0.003; (4) No relevant research;
Barnish and Barran (2020; 56 studies; 1531; 6)	(1) UPDRS−3 motor; (2) TUG; (3) PDQ−39 total score (4) MMSE; (5) MoCA; (6) FAB;	Compared to other exercise: (1) NS (*p =* 0.96); (2) Compared to usually care: NS (*p =* 0.33); (3) Compared to usually care: *p =* 0.0002; (4) (5) (6) No comparable studies for meta–analysis;
Tang et al. (2019; 19 studies; 920; 8)	(1) UPDRS–III; (2) Gait velocity; (3) TUG; (4) BBS; (5) PDQ−39;	Tango vs. control: (1) MD = −9.30, 95%CI = −15.11 to −3.48, *p < * 0.05; (2) MD = 0.13, 95%CI= 0.0748 to 0.1852, *p < * 0.05; (3) MD = 3.15, 95%CI = −5.60 to −0.70, *p < * 0.05; (4) MD = 5.00, 95%CI = 3.74 to 6.26, *p < * 0.05; (5) NS (MD = 2.40, 95%CI = 0.78 to 4.02);
Aguiar et al. (2016; 10 studies; 532; 3)	(1) Walking performance; (2) FOG–Q; (3) Mobility; (4) Balance; (5) Quality of life; (6) Disease severity	This review found weight of the evidence suggests that therapeutic dancing can be beneficial for improving motor performance and balance in people with PD.
Hasan et al. (2022; 14 RCTs; 372; 7)	(1) MDS–UPDRS−1, (2) MDS–UPDRS−2, (3) MDS–UPDRS−3; (4) TUG; (5) BBS; (6) FOG; (7) 6–MWT; (8) Forward velocity (m/s) and Backward velocity (m/s); (9) Mini–BESTest; (10) BDI; (11) AS; (12) PDQ−39; (13) MoCA;	Compared to control: (1) NS (*p =* 0.20); (2) NS (*p =* 0.26); (3) MD = −4.49, 95%CI = −6.78 to −2.21, p = 0.0001 (at 3 months) (4) MD = −1.28, 95%CI = −1.99 to −0.57, *p < * 0.004 (at 3 months); (5) MD = 5.25, 95%CI = 3.8 to 6.7, *p < * 0.00001 (at 3 months); (6) NS (at 3 months); (7) NS (at 3 months, *p =* 0.42) (8) NS (forward velocity: at 3 months); (9) MD= 2.68, 95%CI = 0.82 to 4.54, *p =* 0.005 (at 3 months); (10) NS (at 3 months, *p =* 0.33); (11) MD = −3.37, 95%CI = −5.86 to −0.88, *p =* 0.008 (at 3 months); (12) NS (at 10 weeks, *p =* 0.68; at 3 months, *p =* 0.81); (13) MD= 1.1, 95%CI = 0.36 to 1.85, *p =* 0.004 (at 3 months);
Mandelbaum and Lo (2014; 9 RCTs; 295; 4)	(1) Gait; (2) Balance; (3) Upper Extremity Function; (4) Disability Rating; (5) Falls; (6) Quality of life; (7) Drop-out/Exit Survey; (8) Safety and Tolerability;	This review concluded that studies of dance intervention for PD patients should include an active randomized controlled group, a blinded evaluator, power analysis, minimally important difference, and intention-to-treat analysis.

PD, Parkinson's disease; MoCA, The Montreal Cognitive Assessment; FAB, Frontal Assessment Battery; SDS, Self-rating Depression Scale; BDI, Beck Depression Inventory; AS, Apathy Scale; WMD, Weighted Mean Difference; UPDRS-3, Unified Parkinson's Disease Rating Scale Part-3; HandY, Hoehn-Yahr Score; BBS, Berg Balance Scale; TUG, Timed Up-and-Go; 6MWT,6-minute walking test; PDQ-39, Parkinson's Disease Quality of Life Scale; PAS, Physical Activity Scale; Mini-BESTest, Mini-Balance Evaluation Systems Test; FOG-Q, The Freezing of Gait Questionnaire; FTSST, the Five Times Sit-to-Stand Test; SRT, Sit-and-Reach Test; BST, Back Scratch Test; ABC, Activities-Specific Balance Confidence; FES, Fall-Related Self-Efficacy; FSS, Fatigue Severity Scale; High quality: total score ≥7; moderate quality: total score 4–6; low quality: total score ≤3.

PEDro scores (Collins et al., 2018) (total score/10) were used to grade the quality assessment of the included clinical trials and to compare the scientificity of the experimental design of the included studies. The Assessment of Multiple Systematic Reviews (AMSTAR) tool (total score/11) (Shea et al., 2007) was used to assess the methodological quality of included systematic reviews and meta-analysis. Two reviewers independently assessed the methodological quality of included studies. On the basis of the Consensus Statements (Crossley et al., 2016; Collins et al., 2018), the included studies were graded as low quality, moderate quality or high quality. On the basis of PEDro and AMSTAR scores, low quality was ≤3, moderate quality was between 4 and 6, and high quality was ≥7.

### Data analysis

We reviewed the effects of exercise interventions on motor function, cognitive limitation, psychology, and quality of life in accordance with disease type (i.e., MCI, AD, or PD classification). This process was performed because of differences in disease type, intervention group, and primary outcome assessment in the included studies. The effects of DMT were compared with other exercises, usual care, and treatment involved in the meta-analyses and RCTs in accordance with the clinical features of each disease.

## Results

**Figure 1:**
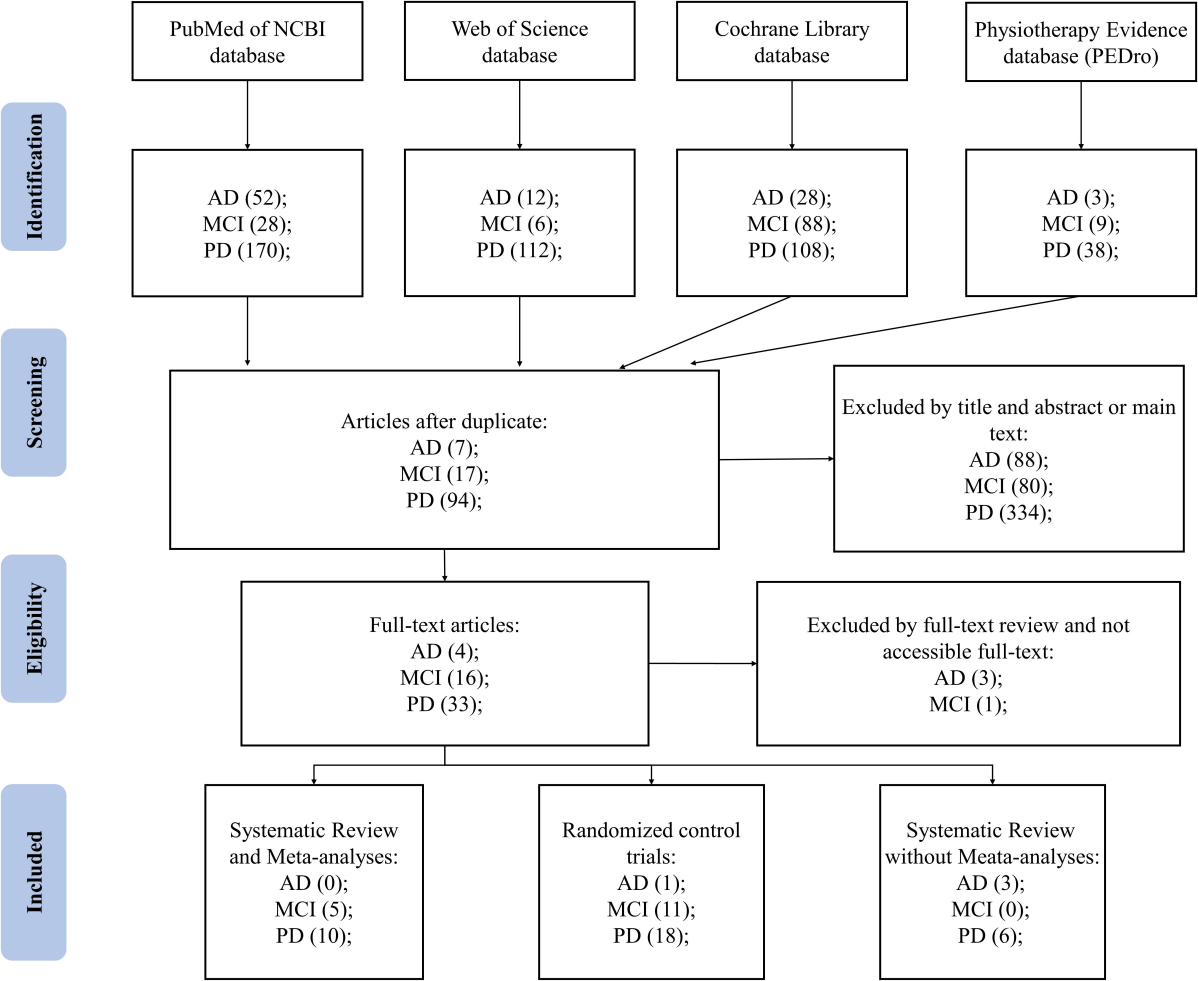
Flowchart of article search, exclusion, and analyses. AD, Alzheimer's disease; MCI, Mild cognition impairment; PD, Parkinson's disease.

A total of 262 papers overlapped (i.e., the same papers were extracted from different search source) out of the 721 papers extracted in the preliminary search. A total of 295 reports were excluded because of the following reasons: (1) the experimental design was not a systematic analysis or RCT; (2) the type of participants included in the study was complex (e.g., included MCI, stroke, etc.); (3) no control group; (4) no comparison or missing results were provided; (5) study was not completed. A total of 130 articles were retained for further research, and 73 articles were excluded for reasons similar to those described above. Thirty-three RCTs and 24 systematic analyses were included. As shown in Figure 1, thirty-three RCTs and 24 systematic analyses were included.

### Dance-movement therapy for mild cognitive impairment

#### Description of mild cognitive impairment

MCI was introduced as the intermediate stage between healthy aging with mild cognitive changes and dementia (Petersen et al., 2014). In accordance with the main symptoms, MCI was divided into amnestic MCI (aMCI) with memory dysfunction predominates and non-amnestic MCI with other cognitive deficit syndrome predominates (e.g., language, visuospatial, executive) (Petersen et al., 2018). Higher prevalence of MCI is related to increased age and lower educational level. Among the 65-year-old population, 16–20% had MCI, with a 5-year cumulative incidence ranging from 22.9 to 30.1%. MCI had a tendency to convert to dementia, with an annual conversion rate of 10% (Zhu et al., 2018). Although the neuropathogenesis of MCI and dementia is still being further explored and without definitive treatment. The population with MCI retains neuroplasticity. Existing studies have found that rTMS (Chou et al., 2020), cognitive therapy (Kirova et al., 2015), aerobic exercise, psychological and social activities (Langa and Levine, 2014; Jongsiriyanyong and Limpawattana, 2018), supplementing nutrition (Eshkoor et al., 2015) and maintaining adequate sleep (Blackman et al., 2021) can prevent the deterioration of the disease to a certain extent. In accordance with the recommendations for the management of MCI, regular physical activity for twice a week was more effective than cognitive impairment drugs. Recent studies demonstrated that dance movement intervention has a positive effect on cognitive function in MCI patients.

#### Effect on global cognitive function

The Mini-Mental State Examination (MMSE), Montreal Cognitive Assessment (MoCA), and Function and Cognitive Assessment Test (FUCAS) were used to assess the global cognitive level of MCI patients. Three high-quality systemic and meta-analysis studies (Chan et al., 2020; Liu et al., 2021; Wu et al., 2021) reported that global cognitive function remarkably increased when older adults with MCI practiced dance compared with the control group [Wu et al. (2021), SMD = 0.54, Z = 3.55, *p* < 0.001; Liu et al. (2021), SMD = 0.73, 95%CI = 0.47 to 0.99, *p* < 0.001; Chan et al. (2020), SMD = 0.48, 95%CI = 0.21 to 0.74]. The meta-analysis results of Wu et al. (2021) showed that the efficacy of DMT on the MMSE is related to the overall duration of intervention. The longer the intervention duration (≥3 months), the better the clinical benefit (Wu et al., 2021). Of the five RCTs that examined the efficacy of DMT in patients with MCI using global cognitive function as the primary outcome, two RCTs reported the positive effect of DMT (Lazarou et al., 2017; Chang et al., 2021). Lazarou et al. (2017) reported that MCI patients who performed dancing for 10 months have improved MMSE (*p* < 0.001) and MoCA (*p* = 0.03) scores compared with usual care. Chang et al. (2021) compared the effects of Chinese square dancing and usual care in MCI patients and reported that the group^*^time effect of MoCA scores is only seen at week 18. Rektorova et al. (2020) showed no remarkable difference in MoCA score between dance practice and life as usual for 24 weeks in MCI patients. Two 12-week RCTs of aerobic dance versus usual care reported no significant between-group differences in MoCA scores in MCI patients (Zhu et al., 2018; Qi et al., 2019). Performing DMT for at least 3 months has positive effects on the global cognitive function of MCI patients.

#### Effect on memory and other cognitive functions

Comprehensive cognitive function assessment includes memory, working memory, attention, executive function, and language. Immediate and delayed recall was commonly used to assess participant's memory ability. Five systematic reviews and meta-analyses confirmed that DMT significantly improved memory ability in MCI patients (Chan et al., 2020; Zhu et al., 2020; Liu et al., 2021; Wu et al., 2021). Among the seven (Cross et al., 2012; Doi et al., 2017; Lazarou et al., 2017; Zhu et al., 2018; Kropacova et al., 2019; Qi et al., 2019; Rektorova et al., 2020) included RCTs, two employed Digit Span Task (DST) (Zhu et al., 2018; Qi et al., 2019), one (Lazarou et al., 2017) used Rey Osterrieth Complex Figure Test copy and delay recall (RAVLT) and Rivermead Behavioral Memory Test-1 (RBMT-1), and others used word memory (Doi et al., 2017) to assess the immediate memory recall. Two studies comparing usual care found that 12 weeks of moderate aerobic dance did not produce a significant positive effect on the DST score in MCI patients (Zhu et al., 2018; Qi et al., 2019). Wechsler Memory Scale (WMS), RAVLT, RBMT-2, and story memory test were used to evaluate the delayed recall. Four RCTs (Zhu et al., 2018, 2022; Kropacova et al., 2019; Qi et al., 2019) with WMS as the primary outcome reported that 12 weeks of aerobic dance had positive effects on delayed recall ability in MCI patients compared with usual care or health education. The RCT of Lazarou et al. (2017) showed that better immediate and delayed recall performance is detected in 10 months of international ballroom dancing compared with the control group (RAVLT: *p* = 0.003; RBMT-1, *p* = 0.004; RBMT-2, *p* = 0.001). The results of Doi et al. (2017) reported that one dance intervention for 0.5 h can improve story memory recall in patients with MCI compared with health education or music. And the finding of Zhu et al. (2022) showed that 12 weeks of aerobic dance could improve episodic memory (β= 0.326, 95%CI = 1.005 to 6.773, *p* = 0.009) and increase the right (β= 0.379, 95% CI = 0.117 to 0.488, *p* = 0.002) and total (β= 0.344, 95% CI = 0.082 to 0.446, *p* = 0.005) hippocampal volumes in the individual with MCI. Remarkable cortical thickening was observed in the right inferior temporal, fusiform, and lateral occipital regions of MCI participants undergoing a 6-month dance intervention (Rektorova et al., 2020). Overall, DMT had significant positive effects in improving the memory ability in MCI patients regardless of the intervention type and duration.

Tables 1–4 show the effects of DMT on executive function, which is assessed by using Trail Making Test, Five-Point Test, Tower of Hanoi, and Rey Osterrieth Complex Figure Test. Two meta-analyses showed (Zhu et al., 2020; Wu et al., 2021) that DMT has remarkable effects on executive function compared with non-dance intervention. Among the five included RCTs with executive function as the primary outcome, three trials supported that DMT can significantly increase the response time and accuracy in MCI patients (Zhu et al., 2018; Kropacova et al., 2019; Qi et al., 2019). The Verbal Fluency F-A-S Test (FAS) and Mississippi Aphasia Screening Test (MAST) are the commonly used language-cognition assessment tools. The results of three meta-analyses demonstrated that DMT positively enhances the verbal fluency in MCI patients (Liu et al., 2021; Wu et al., 2021). Two meta-analyses confirmed that DMT can improve in the visuospatial function of patients with MCI (Chan et al., 2020; Wu et al., 2021).

#### Effect on physical and psychological function

Balance and depression contribute to reduced quality of life in individuals with MCI. The efficacy of DMT is associated with the intervention time to some extent. Aerobic dancing for 12 weeks did not improve the balance in MCI patients compared with usual care (Qi et al., 2019). Participants in the intervention group showed remarkable time^*^group effect in improving balance, depression, and quality of life compared with usual care after 18 weeks of Chinese square dancing (Chang et al., 2021). Compared with music, 0.5 h dance intervention showed significant improvement in depressive symptoms in MCI patients over the first 3 days (Cross et al., 2012). Two RCTs reported that more than 8 weeks of dance training provided cardioprotective benefits in patients with MCI (Aguiñaga and Marquez, 2017; Ammar et al., 2021). Therefore, DMT is an efficient rehabilitation strategy to promote the wellbeing of patients with MCI.

### Dance-movement therapy for Parkinson's disease

#### Description of Parkinson's disease

PD is a chronic, progressive, and disabling ND. As the second most common ND, PD affects more than 6 million older adults worldwide. The gold standard for its diagnosis is the pathological changes in Lewy bodies and the degeneration of substantia nigra pars compacta (SNpc) at autopsy (Dickson et al., 2009). In the misfolded state, α-synuclein becomes insoluble and aggregates within the cell bodies (Lewy bodies) and processes (Lewy neurites) of neurons to form intracellular inclusions (Polymeropoulos et al., 1997; Wong and Krainc, 2017). This condition is due to the death of the dopaminergic neurons of SNpc in the early stage, resulting in dopamine deficiency in the basal ganglia and typical Parkinson's motor dysfunction (Dickson et al., 2009). The symptoms of PD include slow movements, muscle stiffness, rest tremor, postural instability, and gait disturbances. PD involves non-motor features, including psychiatric disturbances, cognitive impairments, olfactory disturbances, sleep disturbances, autonomic dysfunction, pain, and fatigue. It affects physical, mental, emotional, and social functioning, which can have a profound effect on the quality of life (Sharp and Hewitt, 2014). Despite the recent advances in gene and drug therapy, no disease treatments that can improve PD. Current research suggests that exercise increases brain volume, promotes the activation of a wide range of brain regions, and produces neuroprotective effects that allow the brain to repair itself. Exercise-induced behavioral recovery and increased dopamine synthesis and release in PD rats have been reported in several animal studies. A number of studies have confirmed that active intervention for dance therapy may be the most suitable exercise intervention for PD patients due to the musical rhythm background and high patient compliance.

#### Effects on motor symptoms

Sixteen systematic analyses (Mandelbaum and Lo, 2014; Sharp and Hewitt, 2014; Lötzke et al., 2015; Shanahan et al., 2015; Aguiar et al., 2016; Dos Santos Delabary et al., 2018; Kalyani et al., 2019; Tang et al., 2019; Zhang Q. et al., 2019; Barnish and Barran, 2020; Berti et al., 2020; Carapellotti et al., 2020; Hidalgo-Agudo et al., 2020; Emmanouilidis et al., 2021; Ismail et al., 2021; Hasan et al., 2022), 18 RCTs (Hackney et al., 2007; Hackney and Earhart, 2009, 2010; Hashimoto et al., 2015; Rios Romenets et al., 2015; Hulbert et al., 2017; Shanahan et al., 2017; Lee et al., 2018; Michels et al., 2018; Poier et al., 2019; Solla et al., 2019; Frisaldi et al., 2021), and 62 clinical trials investigated the effects of dance on Parkinson's symptoms (Tables 1–4, Supplementary Tables 1, 2). Most studies (11/13 systematic analyses and all RCTs) examined the effects of dance exercise therapy in PD patients using exercise parameters as the primary outcome measure. Movement Disorder Society Unified Parkinson's Disease Rating Scale (MDS-UPDRS) Section Introduction–Result was applied to evaluate the global disease severity condition of patients with PD. Cognitive and psychological symptoms were measured by MDS-UPDRS-1. MDS-UPDRS-2 covered the activities of activity daily living. MDS-UPDRS-3 evaluated motor functions, such as tremor, bradykinesia, postural instability, rigidity, and gait. Six meta-analyses (Sharp and Hewitt, 2014; Lötzke et al., 2015; Dos Santos Delabary et al., 2018; Kalyani et al., 2019; Ismail et al., 2021; Hasan et al., 2022) reported higher MDS-UPDRS-3 scores on PD patients who had DMT compared with the non-dance group. The DMT group obtained significantly better MDS-UPDRS-3 scores compared with the no treatment or other exercise groups (SMD = −10.73, 95%CI = −15.05 to −6.16, *p* = 0.004), and standard or other physical therapy (MD= −6.91, 95%CI = −9.97 to −3.84, *p* < 0.05) (Sharp and Hewitt, 2014; Ismail et al., 2021). More than half of trials with MDS-UPDRS-3 as the primary outcome measure demonstrated that dance intervention improved the overall motor performance in PD patients (Duncan and Earhart, 2012, 2014; Volpe et al., 2013; Hashimoto et al., 2015; Lee et al., 2018; Solla et al., 2019; Frisaldi et al., 2021). In a 2-year RCT of dance intervention, a significant time-group interaction effect was found on MDS-UPDRS-3 in PD patients (F _[2, 8]_ = 17.59; *p* < 0.001) (Duncan and Earhart, 2014). DMT can be a long-term intervention for the management and rehabilitation of patients with PD.

Timed Up and Go test (TUG) (Hackney et al., 2007; Hackney and Earhart, 2009, 2010; Volpe et al., 2013; Duncan and Earhart, 2014; Hashimoto et al., 2015; Rios Romenets et al., 2015; Michels et al., 2018; Solla et al., 2019; Tang et al., 2019; Frisaldi et al., 2021), the mini-BESTest (Lötzke et al., 2015), and BBS (Hackney et al., 2007; Hackney and Earhart, 2009, 2010; Volpe et al., 2013; Hashimoto et al., 2015; Lee et al., 2015, 2018; Michels et al., 2018; Solla et al., 2019; Tang et al., 2019) were used to assess the participant's balance. Six-minute walk test (6 MWT) (Hackney and Earhart, 2009, 2010; Duncan and Earhart, 2012; Volpe et al., 2013; Shanahan et al., 2017; Solla et al., 2019; Frisaldi et al., 2021) and the Freezing of Gait Questionnaire (FOG-Q) (Hackney et al., 2007; Hackney and Earhart, 2009; Duncan and Earhart, 2012, 2014; Volpe et al., 2013; Rios Romenets et al., 2015) were the commonly used gait evaluation methods. Eight meta-analyses showed that DMT had positive effects in the balance and gait of PD patients compared with the non-dance treatment group (Sharp and Hewitt, 2014; Lötzke et al., 2015; Dos Santos Delabary et al., 2018; Kalyani et al., 2019; Tang et al., 2019; Hidalgo-Agudo et al., 2020; Ismail et al., 2021; Hasan et al., 2022). The meta-analysis results of Ismail et al. (2021) reported that DMT is significantly more effective than usual care or other physical therapies in terms of balance and gait (BBS, MD = 8.42, 95%CI = 3.68 to 13.17, *p* < 0.05, at 3 months; TUG, MD= −1.16, 95%CI = −2.17 to −0.15, *p* < 0.05; FOG-Q, MD = −0.39, 95%CI = −2.99 to 2.24, *p* < 0.05; 6 MWT, MD = 238.80, 95%CI = 157.99 to 319.61, *p* < 0.05). The meta-analysis results showed that Tango is more advantageous in improving balance and gait speed in individuals with PD by comparing the effects of Tai chi, Qigong, yoga, and resistance training on PD in terms of gait velocity, TUG, and BBS (Tang et al., 2019). The meta-analysis finding of Shanahan et al. (2015) reported that two 1 h DMTs per week for 10 to 13 weeks can provide potential positive effects on endurance, balance, and disease management. However, the effect of DMT on BBS is controversial. More than half (5/9) of RCTs using BBS as the primary outcome did not observe significant difference between the DMT group and no-treatment, usual care or other exercise groups (Hackney et al., 2007; Volpe et al., 2013; Lee et al., 2018; Qi et al., 2019; Chang et al., 2021). The RCT results of Hulbert et al. (2017) of DMT for 27 mild to moderate PD patients, 3D motion analysis, and clinical measurements found that head, pelvis, and foot movements during turning were affected by dancing with tighter coupling of body segments. Compared with conventional treatment, 20 dance sessions resulted in longer head latency (*p* = 0.008) and increased pelvic rotation (*p* = 0.036) in PD patients when turning around (Hulbert et al., 2017). As of June 2020, the finding of Emmanouilidis et al. (2021) supported that DMT can remarkably improve the disease condition, mobility, balance, and gait in PD patients in the short term.

Of the 18 RCTs, 9 studied Tango, and the rest included Irish dance, Sardinian dance, double ballroom, Latin American dance, and Turo. Tango is more suitable for PD patients than other dance styles due to its characteristic of “frequent movement starts and stops, a series of changes in speed and rhythm.” However, none of the trials had a dance therapist assisting during the intervention. The total intervention duration ranged from 15 h in 5 weeks to 192 h in 96 weeks. Taking 12 weeks as the dividing line, 8 short-term (Hackney and Earhart, 2010; Lee et al., 2015, 2018; Hulbert et al., 2017; Shanahan et al., 2017; Michels et al., 2018; Poier et al., 2019; Frisaldi et al., 2021) interventions (total intervention duration <12 weeks) and 11 long-term (Hackney et al., 2007; Hackney and Earhart, 2009; Duncan and Earhart, 2012, 2014; Foster et al., 2013; Volpe et al., 2013; Hashimoto et al., 2015; Rios Romenets et al., 2015; Solla et al., 2019; Young et al., 2019; Moratelli et al., 2021) interventions (total intervention duration ≥12 weeks) were found. The attendance of participants in DMT group was not reported in 9 of the 18 RCTs assessing various DMT in the population with PD. In RCTs studies that have reported attendance rates, participants in the DMT group had an average attendance rate of greater than 70% and greater compliance than the traditional exercise or usual care control group (Hackney et al., 2007; Hackney and Earhart, 2010; Duncan and Earhart, 2012; Volpe et al., 2013; Rios Romenets et al., 2015; Shanahan et al., 2017; Michels et al., 2018; Solla et al., 2019; Moratelli et al., 2021). Two RCTs reported that participants in the dance group continued to take dance lessons after completing the entire experiment (Hackney et al., 2007; Rios Romenets et al., 2015). In accordance with the criteria of ASMART and PEDro, all RCTs were of moderate or above quality (>4 score).

#### Effects on cognitive deficit

Four systematic reviews and meta-analyses explored the efficacy of DMT on the overall cognitive level of PD patients, where three of them confirmed that the DMT group is better than the control group (Kalyani et al., 2019; Zhang Q. et al., 2019; Hasan et al., 2022). The meta-analysis of Zhang Q. et al. (2019) reported that DMT had remarkable difference in executive function compared with the non-dance group (WMD = 1.17, 95%CI = 0.39 to 1.95, *p* = 0.003). Cognitive dual-task evaluation by dual-task TUG showed that PD patients in the dance groups had better improvement (SMD = −0.85, 95% CI = −1.50 to −0.21, *p* < 0.05) (Kalyani et al., 2019). In four (Rios Romenets et al., 2015; Solla et al., 2019; Frisaldi et al., 2021; Moratelli et al., 2021) RCTs, DMT improved the overall cognitive function in PD patients compared with usual care, which were measured by MoCA. Two RCTs (Hashimoto et al., 2015; Solla et al., 2019) reported that 12-week dance program showed significant differences in PD patients in terms of task switching and mental flexibility (measured by the Frontal Assessment Battery at bedside (*p* = 0.001), and the Mental Rotation Task (response time: *p* < 0.001).

#### Effects on mood and quality of life

The clinical evaluation tools commonly used for depression and apathy include the Beck Depression Inventory and the Apathy Scale. As of April 2020, no meta-analysis showed that dance can improve the depression in PD patients. More than half (Rios Romenets et al., 2015; Poier et al., 2019; Frisaldi et al., 2021) of the RCTs examining the effects of dance on depression showed the same results. The results of two meta-analysis considering the effect of DMT on apathy in PD patients were controversial. Compared with non-dance intervention, the meta-analysis results of Zhang Q. et al. (2019) reported no positive effects in the depression and apathy of PD patients in the dance group. The meta-analysis result of Hasan et al. (2022) showed that the reduction in apathy scores was greater in the DMT group than in the no-treatment group at 3 months (MD = −3.37, 95%CI = −5.86 to −0.88, *p* = 0.008). In a 12-week Sardinian dance RCT, the significant time^*^group interactions for depression (*p* < 0.001) and apathy (*p* = 0.016) were observed in the dance group (Solla et al., 2019).

Whether DMT can improve the Quality of Life Scale-39 item (PDQ-39) in PD patients remains controversial. Compared with the non-dance group, more than half of the meta-analyses (Dos Santos Delabary et al., 2018; Tang et al., 2019; Hasan et al., 2022) and RCTs (Shanahan et al., 2017; Poier et al., 2019; Frisaldi et al., 2021; Moratelli et al., 2021) reported no remarkable differences in the DMT group. The meta-analysis result of Sharp and Hewitt (2014) reported that DMT can have short-term clinically meaningful benefits in PD patients (PDQ-39, MD= −4.00, 95%CI= −7.13 to −0.87, *p* = 0.01). Compared with usual care, DMT provided PD patients with a large number of new social activities (*p* = 0.003) (Foster et al., 2013) and better emotional wellbeing (*p* = 0.039) (Poier et al., 2019).

### Dance-movement therapy for Alzheimer's disease

AD is a collective term for a variety of progressive degenerative brain syndromes that affect cognition, behavior, mood, and social function. As the seventh leading cause of death, AD affects more than 50 million people worldwide (Hodson, 2018; Estimates, 2022). In accordance with the estimation of the Alzheimer's Association, individuals with AD will reach 115.4 million by 2050 due to the increasing population of older adults. AD has become an increasing issue of public health importance as people live longer and many countries have aging populations.

Three above-moderate quality systematic analyses (Karkou and Meekums, 2017; Mabire et al., 2019; Ruiz-Muelle and López-Rodríguez, 2019), 1 low RCT (Low et al., 2016), and 1 review (Bennett et al., 2021) reported the role of dance in patients with AD. All three systematic analyses looked at motor symptoms, cognitive impairment, mood, and quality of life in AD patients, but their focus varied. The analyses of Karkou and Meekums (2017) found that no professional dance therapists are involved in the RCTs of AD patients. The finding of (Ruiz-Muelle and López-Rodríguez, 2019) supported that DMT has potential positive effects on the recovery of cognitive function and the improvement of the quality of life in patients with AD. The results of Mabire et al. (2019) confirmed the positive effect of dance in improving the symptoms of AD patients and provided nine practical recommendations for implementing dance intervention, including intervention dosage, precautions. This review suggests that dance has the potential to influence motor symptoms, cognitive deficits, mood, and quality of life of AD patients. However, the evidence is weak, and further research is warranted. The therapeutic waltz for 10 weeks can significantly improve the concentration and communication with others in patients with AD (Hamill et al., 2012). Physical exercise, including dancing, lowered the MMSE score for AD patients (Hernandez et al., 2010). In two studies, a 10-min elderly clown intervention for twice a week in 12 weeks was provided for moderate to severe elderly patients with AD by using improvisation, humor, empathy and expressions of song, instrument or dance) Neuropsychiatric Inventory-Nursing Home version. The score of these patients decreased significantly (t = −2.58, *p* = 0.02) compared with the conventional treatment group, thereby improving the quality of life of AD patients and reduced the burden on nursing staff (Kontos et al., 2016).

## Discussion

### Benefits of dance-movement therapy on neurodegenerative diseases

Combining the results of Karkou and Meekums (2017), this review summarized the characteristics of dance as (a) repetitive body movement exercise; (b) musical and rhythmic auditory stimulant; (c) creativity and “motor mirroring.” DMT is widely recommended for the brain health. Currently, studies on the efficacy of DMT in the intervention of ND mainly focus on the population with MCI, PD or AD. Our systematic review suggested that DMT had positive effects on cognitive function in MCI and motor function in PD. Controversy remains on the effect of dance interventions in improving the mood and quality of life in ND patients. This condition may be related to the stage of a specific ND, intervention method, and the duration of the intervention. These findings were based on 25 comprehensive systematic reviews and 33 RCTs. Future research on the effects of DMT on AD requires scientific design, large sample size, long-term comprehensive intervention, and clear reporting standards. Overall, DMT can be a safe, beneficial, and easily accessible adjunctive treatment for individuals with MCI, PD, or AD.

### Mechanisms of dance therapy for neurodegenerative diseases

**Figure 2:**
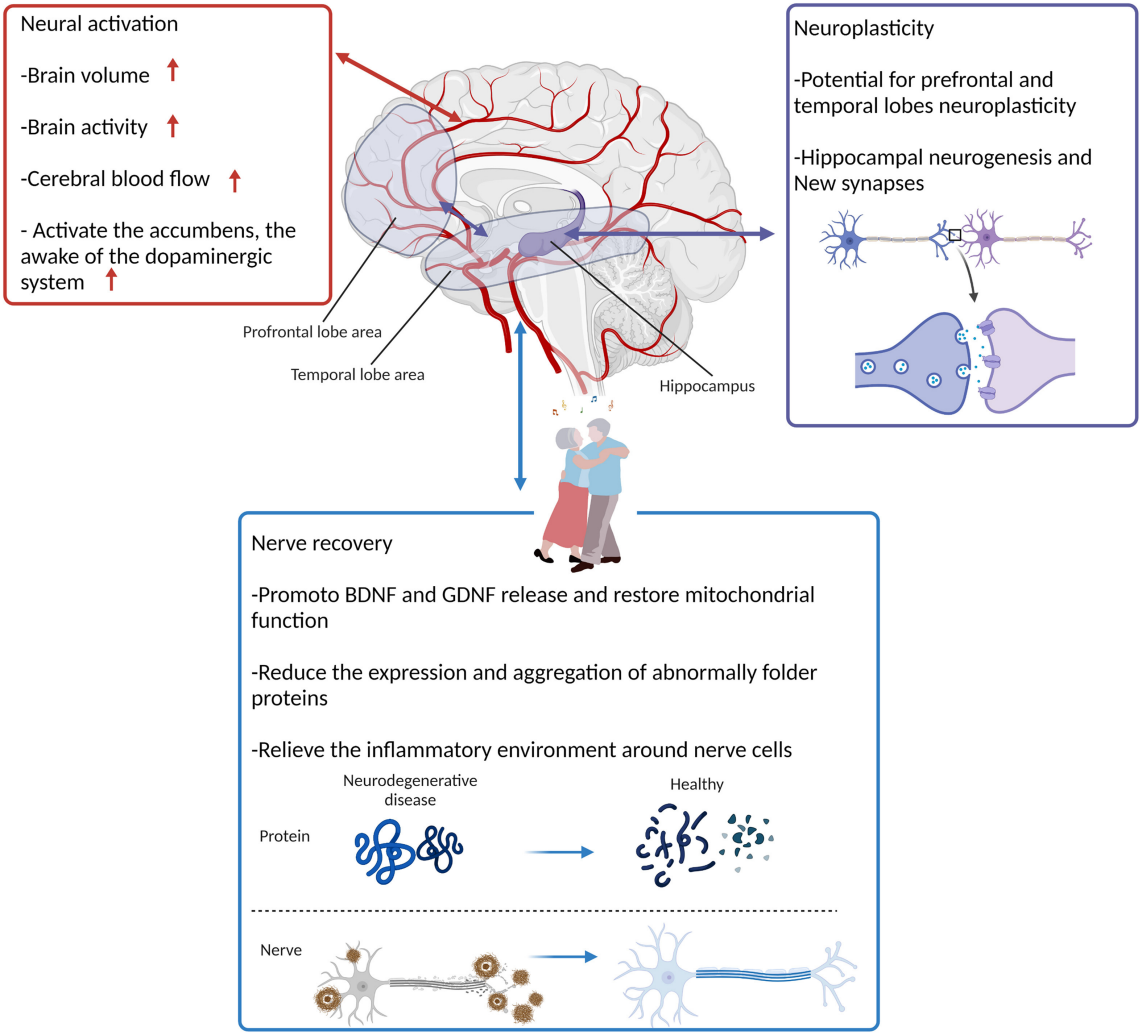
Possible neurobiological mechanisms for the rehabilitative effect of DMT. BDNF, Brain-derived neurotrophic factor; GDNF, Glial derived neurotrophic factor. Created with BioRender.com.

No appropriate animal experimental model is available, and animal experiments related to dance are lacking due to the artistic creativity of dance. Clinical trials involving dance still focus on behavioral outcomes. This review uses the mechanistic framework of Sihvonen et al. (2017) on the intervention of music in NDs and attempts to combine the characteristics of dance to explain the mechanisms behind behavioral outcomes in clinical trials (Figure 2).

#### Neural activation, neuroplasticity, and nerve recovery

The atrophy of brain function and structure in NDs precedes the typical symptoms. On the basis of the result of structural magnetic resonance imaging, typical Alzheimer's brain atrophy begins in the medial temporal lobe to the lateral temporal lobe and then to the parietal cortex. Neuroimaging studies reported that a network of brain regions, including the superior temporal gyrus, superior parietal lobule, frontopolar cortex, and middle temporal gyrus, is involved in dancing (Brown et al., 2006; Cruz-Garza et al., 2014). The activation of corresponding brain regions may be related to DMT involving repetitive body movement exercise coupled with auditory feedback and extensive cognitive processing. Concomitant with angiogenesis and endothelial cell proliferation induced by exercise, blood flow in the middle cerebral artery increases and maintains these effects over time (van Praag et al., 2005; van Praag, 2009). This autoregulation induced by dance has protective benefits against the progression of symptoms in ND. The effect of functional improvement in the population with PD depends on the subjective motivation of dance participation and the duration of the intervention.

Studies have reported that patients with mild to moderate AD still retain motor learning function, which can be due to the potential for neuroplasticity (Sabe et al., 1995; Rösler et al., 2002). The research of Sihvonen et al. (2017) supported dance as active music-based neurological rehabilitation inducing similar structural and functional neuroplastic changes reported in ND patients who received DMT. Existing animal studies confirmed that physical activity can induce hippocampal plasticity, which is associated with hippocampal neurogenesis (van Praag et al., 1999b; van Praag, 2008), form new synapses (van Praag et al., 1999a, 2005; Stranahan et al., 2006), and modulate the release and utilization of neurotransmitters (Vaynman et al., 2004; Vaynman and Gomez-Pinilla, 2005) to rebuild and remodel damaged neural networks. Compared with the control group, 8-week treadmill training in PD rats increased the potential of dopamine D2 receptor binding, suggesting that the efficacy of exercise in improving PD symptoms is associated with neuroplasticity in the dopaminergic pathway (Fisher et al., 2013). When participants performed a dance exercise, a “mirror neuron system” was activated to learn and correct dance movements by observed and imagined performing either familiar or novel movement sequences. Premotor and parietal cortices are considered the key cortices for the “mirror neuron system.” Prefrontal lobe and temporal lobe activity increases, and its regional neuroplasticity is enhanced by receiving a steady stream of dance movement image stimuli, action commands, and musical awakening. Some RCTs reported improvement in the overall cognitive function level after dance training in PD patients. Others studies have provided further evidence for auditory- and motor-related neuroplasticity following community dance therapy and rhythm-movement therapy in PD patients.

Animal research results have shown that physical activity has neurorestorative and neuroprotective effects, and supports synapse formation and angiogenesis by modulating brain-derived neurotrophic factor and glial-derived neurotrophic factor, inhibiting oxidative stress, and improving mitochondrial function (Tajiri et al., 2010; Correale et al., 2017). Treadmill exercise can increase the sirtuin-1 expression, which results in increased mitochondrial biogenesis and decreased oxidative stress (Koo and Cho, 2017). The result of Wu et al. (2018) reported that exercise can inhibit apoptosis-like cell death of neurons in hippocampal area, significant loss of presynaptic/postsynaptic markers, release of proinflammatory cytokines, and oxidative damage in AD rats caused by streptozotocin. Exercise can reduce hippocampal volume atrophy (Tarumi et al., 2019), the expression of α-synuclein in PD rats, and deposition of β-amyloid in the hippocampus of AD rats (Zhang X. et al., 2019; Pena et al., 2020; Wu et al., 2020). Co-stimulation of music and physical activity can provide participants an effect similar to the enriched environment used in animal studies, contributing to the recovery of patients with NDs at the behavioral and neurobiological levels (Baroncelli et al., 2010; Sihvonen et al., 2017).

#### Activation of reward, arousal, and emotion networks

Music and exercise can activate the dopaminergic mesolimbic system, which is involved in the regulation of movement, reward mechanisms, emotion, and cognition. The nucleus accumbens, a key part of the brain's reward system, can be activated by strong emotional responses to music, regulating the dopaminergic mesolimbic system and promoting increased dopamine secretion (Salimpoor et al., 2011). Music-induced increases in parasympathetic activity, lower serum cortisol levels, and inhibition of cardiovascular stress responses have been suggested as the possible underlying mechanisms to explain the cognitive-emotional gains induced by music stimulations in patients with NDs (Salimpoor et al., 2011; Karkou and Meekums, 2017). Physical activity can modulate an intensive increase in dopamine concentration (Kami et al., 2018; Di Liegro et al., 2019). The therapeutic efficacy of physical activity in ND patients includes increasing endorphin levels (Remy et al., 2005; Politis et al., 2010) and attenuation of the hypothalamic pituitary-adrenal axis response to stress (Mayberg et al., 1990; Feldmann et al., 2008). Increased levels of extracellular dopamine secretion may partially explain the cognitive-emotional gains caused by elevated levels of physical activity in patients with ND. A regular, moderate level of physical activity corresponds to a good mental state (Wen et al., 2016). Several clinical studies have reported that physical activity leads to positive emotions associated with mastery and self-efficacy (Leentjens, 2004; Sheng et al., 2014; Lou et al., 2015).

Dancers engage in social interaction and emotional expression through choreographed or improvised body language in specific linguistic and cultural contexts, which may be what differentiates DMT from most physical activities. For example, people perform circle dances around a bonfire to express the joy of a good harvest. And DMT challenges the ability of cognition and balance beyond traditional exercise mode. During the dance programme, participants were required to engage in working memory, attentional control, and multitasking to integrate newly learned and previously learned dance moves, stay in rhythm with the music, and bypass others on the dance floor (Foster et al., 2013). Age-related changes associated with diminished emotional perception may contribute to states of depression or apathy (Alexopoulos, 2005). The results of (Hashimoto et al., 2015) reported 12-week incorporated strategies-based dance can significantly improve the overall disease status, balance, gait, cognitive function, and negative emotions of apathy and depression in PD patients compared with PD exercise (Hashimoto et al., 2015). In addition, participants were able to engage in a certain degree of emotional interaction and catharsis regardless of whether they were actively or passively watching the dance performance. When people watch or perform sad art performances, the emotional resonance it evokes promotes the release of prolactin, which leads to a sense of belonging, comfort, and joy (Christensen et al., 2017).

Social cognition is defined as the ability to interpret and predict the behavior of others based on their beliefs and intentions, and to interact in complex social environments and relationships (Baron-Cohen, 2000). Memory, decision-making, attention, motivation, and mood all increase significantly when socially relevant stimuli trigger behavior (Adolphs, 2009). The findings suggest that even during normal cognitive aging, social cognition declines with age, in part independent of more general cognitive functions. The decline in social cognition in the elderly may be related to the decline observed in neurodegeneration of age-related diseases (Kemp et al., 2012). The study of Foster et al. (2013) reported that PD participants in the dance group had significantly increased social engagement, leisure and social activities compared to the usual living control group, returning most of their activities since the onset of the disease. Compared to traditional exercise, dance classes have additional features that are beneficial to participation. For example, in a pair or multi-person dance, the leader needs to initiate movements and movement plans, while the follower needs to understand and appropriately respond to the leader's instructions. These challenges may further enhance the ability to perform on a daily basis and lead to an increase or maintenance of engagement. The social interaction, social support, and emotional exchange generated by dance class may be beneficial for older people with social cognitive decline. On a personal level, dancing interactions with others may help participants challenge themselves more freely in the complexity and difficulty of dance moves, thereby increasing self-efficacy, which translates into motivation for social activity and social engagement. This may also be one of the reasons why the participants resumed most of their pre-onset activities (Foster et al., 2013).

A distinctive feature of dance therapy is its creativity. Participants immerse themselves in the stimulation of music and rhythm, and engage their emotions in the physical activity that ensues rather than simply learning dance steps. Due to neurocognitive demands, the more complex the motor task, the greater its impact on neuroplasticity. Dance therapists encourage participants to choose or create their own preferred dance moves. This creative and improvised movement engages participants in the non-verbal expression of hard-to-express thoughts and emotions, not only providing opportunities to interact with others, but also helping to promote healthy communication and restore mental balance, which may lead to better emotional and social life (Quinones and Kaddurah-Daouk, 2009). Research has shown that individuals receiving creative activity interventions show significant increases in overall health, quality of life, and physical wellbeing, which in turn have an impact on overall wellbeing (Archer et al., 2015).

#### Activation of alternative or spared neural networks

Therapists often utilize a musical metronome to assist PD patients in the gait starting and walking stages. Sihvonen et al. (2017) speculated that rhythmic entrainment can activate alternative networks in patients with motor system dysfunction. Rhythmic entrainment timed the movement to regular musical beats, enhancing the connectivity between the auditory and motor systems. Bypassing motor system dysfunction and the rhythmic auditory system are used to cue movement execution.

In daily life, a familiar musical melody can recall the memories and emotionally respond. The caudal anterior cingulate gyrus and the ventral pre-supplementary motor area are the key regions for the encoding of musical memory. A neuroimaging study of AD patients found that regions associated with musical memory showed essentially minimal cortical atrophy and less disturbance of glucose metabolism compared with other regions of the brain. This finding provides a potential explanation for the improvement of apathy, depression, and other affective disorders in patients with NDs by musical activity.

### Limitations and future directions

The viewpoints of this review remain limited because of the following reasons. The sample sizes of included RCTs are small, thereby restricting the generalizability of results to the population in the early stages of ND. Most studies included patients with mild to moderate disease, and the findings cannot be generalized to end-stage disease patients. The efficacy of DMT may vary because of different disease stages. Many studies did not control the effects of levodopa or other PD drugs. Furthermore, the different types of DMT and how these make differentially affect mood, physical and cognitive function in various NDs require further exploration. Only studies written in English were included, which may lead to publication bias. Few clinical studies were reported on DMT in the treatment of AD (3 systematic analyses and 1 RCT). These studies had small sample sizes and insufficient follow-up with hidden allocation and blinding. Current studies do not provide sufficient evidence to explain the whole therapeutic mechanisms of DMT. Additional high-quality, large-scale intervention studies and multimodal studies combining behavioral outcome measures with neuroimaging and neuroendocrine markers are needed to elucidate the effects and mechanisms of dance exercise therapy on NDs.

## Conclusions

The proportion of the elderly population in the world continues to increase, and the loss of independence of the elderly due to NDs leads to an increased social burden. Thirty-three RCTs and 24 systematic analyses reported the effects of DMT in NDs. DMT had remarkable effects on the condition, balance, and gait in PD patients worldwide. DMT significantly improved the cognitive function, memory, and executive function for patients with MCI. However, data are insufficient to fully demonstrate that DMT has a positive effect in patients with AD. Future research on the effects of DMT on AD requires scientific design, large sample size, long-term comprehensive intervention, and clear reporting standards.

## Data availability statement

The original contributions presented in the study are included in the article/Supplementary material, further inquiries can be directed to the corresponding author/s.

## Author contributions

X-QW had substantial contributions to the conception of the study. X-QW and J-JZ designed this systematic review. C-CW and H-YX conduct the research. C-CW wrote the original draft of the manuscript. X-QW, J-JZ, H-YX, and C-CW participated in the revision of the draft. All authors read and approved the final submitted version.

## Funding

This study was supported by the National Natural Science Foundation of China (81871844), Shuguang Program supported by Shanghai Education Development Foundation and Shanghai Municipal Education Commission (18SG48), the Science and Technology Commission of Shanghai Municipality (grant numbers 19080503100 and 21S31902400), the Fok Ying-Tong Education Foundation of China (grant number 161092), the Talent Development Fund of Shanghai Municipal (grant number 2021081), the Shanghai Clinical Research Center for Rehabilitation Medicine (grant number 21MC1930200), and the Shanghai Key Lab of Human Performance (Shanghai University of Sport) (grant number 11DZ2261100), as well as by the Shanghai Frontiers Science Research Base of Exercise and Metabolic Health.

## Conflict of interest

The authors declare that the research was conducted in the absence of any commercial or financial relationships that could be construed as a potential conflict of interest.

## Publisher's note

All claims expressed in this article are solely those of the authors and do not necessarily represent those of their affiliated organizations, or those of the publisher, the editors and the reviewers. Any product that may be evaluated in this article, or claim that may be made by its manufacturer, is not guaranteed or endorsed by the publisher.
