# Supplementary material for: Dance movement therapy for neurodegenerative diseases: A systematic review
Source: Front Aging Neurosci. 2022 Aug 8;14:975711. doi: 10.3389/fnagi.2022.975711 (PMC9394857; doi:10.3389/fnagi.2022.975711)
Supplement: Supplementary file 1 [file Data_Sheet_1.docx]

**Supplementary search strategy**

Search terms adapted from the PubMed database, Web of Science Collection database, Cochrane library, and Physiotherapy Evidence Database (PEDro).

**The search strategy for four databases of dance-base movement interventions in Mild Cognition impairment.**

In the PubMed database:

“(dance[Title/Abstract]) AND (Cognition impairment [Title/Abstract])”;

In the Web of Science Collection database:

“(Title= dance) AND (Title= Cognition impairment)”

In the Cochrane library:

“(Title Abstract Keyword= dance) AND (Title Abstract Keyword= Cognition impairment)”

In the Physiotherapy Evidence Database (PEDro):

“Abstract & Title= dance and Cognition impairment”

**The search strategy for four databases of dance-base movement interventions in Parkinson's disease.**

In the PubMed database:

“(dance[Title/Abstract]) AND (Parkinson's disease [Title/Abstract])”;

In the Web of Science Collection database:

“(Title= dance) AND (Title= Parkinson's disease)”

In the Cochrane library:

“(Title Abstract Keyword= dance) AND (Title Abstract Keyword= Parkinson's disease)”

In the Physiotherapy Evidence Database (PEDro):

“Abstract & Title= dance and Parkinson's disease”

**The search strategy for four databases of dance-base movement interventions in Alzheimer's disease.**

In the PubMed database:

“(dance[Title/Abstract]) AND (Alzheimer’s disease[Title/Abstract])”;

In the Web of Science Collection database:

“(Title= dance) AND (Title= Alzheimer’s disease)”

In the Cochrane library:

“(Title Abstract Keyword= dance) AND (Title Abstract Keyword= Alzheimer’s disease)”

In the Physiotherapy Evidence Database (PEDro):

“Abstract & Title= dance and Alzheimer’s disease”;

**Supplementary Table 1:** Characteristics of randomized controlled trials assessing various Dance-based movement interventions in patients with mild cognitive impairment and Alzheimer’s disease.

| Experimental group vs control group | Participants (n) and condition | Dance therapist involved | Blinding | Quality (0-10 score) |  |
| --- | --- | --- | --- | --- | --- |
| **Mild Cognitive Impairment** |  |  |  |  |  |
| Aerobic dance routine vs usual care (Zhu et al., 2018) | 60 MCI participants (male/female=24/36; mean age= 69.65±7yr); | No | Single (assessor) | High  (7) |  |
| Aerobic dance vs health education (Zhu et al., 2022) | 68 MCI participants (male/female=27/41; mean age= 70.67±7.18yr); | No | Single (assessor) | Moderate  (6) |  |
| Dance vs a life as usual (Rektorova et al., 2020) | 62 MCI participants (male/female=17/45; mean age= 67.6±5.8yr; MoCA mean score= 26.74±2.6; year of education= 14.75±3.1yr); | No | Single (assessor) | Moderate  (4) |  |
| Specially designed moderate-intensity aerobic dance vs usual care (Qi et al., 2019) | 32 MCI participants (male/female=9/23; mean age= 69.85±7.15yr; MoCA mean score= 22.75±1.9; year of education= 10.05±2.65yr); | No | No | Moderate  (5) |  |
| Participants familiarized dance vs usual care (Lazarou et al., 2017) | 129 MCI participants (male/female=28/101; mean age= 68.77±5.73yr; MoCA mean score= 28.01±2.80; year of education= 10.86±4.58yr); | No | Single (assessors) | Moderate  (6) |  |
| Dance vs life-as-usual (Kropacova et al., 2019) | 99 MCI participants (male/female=23/76; mean age= 66.8±10.1yr; year of education= 14.66±2.83yr); | No | No | Moderate  (6) |  |
| Dance, music, and health education (Doi et al., 2017) | 201 MCI participants (male/female=97/104; mean age= 75.97±4.53yr; year of education= 11.63±2.63yr); | No | No | High  (8) |  |
| Dance vs music (Cross et al., 2012) | 100 MCI participants (male/female=47/53; mean age= 76.44yr; Depression mean scale= 17.13±4.78; Memory mean scale= 6.82±2.9); | No | No | Moderate  (5) |  |
| Chinese square dance vs usual care (Chang et al., 2021) | 109 MCI participants (male/female=47/53; mean age= 76.56±3.60yr; MoCA mean score= 43.09±6.49; year of education= 8.73±2.05yr); | No | Single (assessors) | Moderate  (5) |  |
| Fitness-dance vs life as usual (Ammar et al., 2021) | 12 MCI participants (male/female=6/6; mean age= 73 ± 4.40yr); | No | No | Moderate  (4) |  |
| BAILAMOS vs waitlist control (Aguiñaga et al., 2020) | 21 MCI participants (mean age= 75.4±6.3yr; mean MMSE score= 22.4±2.8; year of education= 8.73±2.05yr); | No | No | Moderate  (4) |  |
| **Alzheimer’s disease** |  |  |  |  |  |
| dance groups vs music appreciation and socialization groups (Low et al., 2016) | 18 AD participants, Suitability of measures (mean age = 85.3±6.2yr); | No | No | Low  (2) |  |

MCI= Mild cognitive impairment; yr= year; AD= Alzheimer’s disease; High quality: total score ≥7; moderate quality: total score 4–6; low quality: total score ≤3.

**Supplementary Table 2:** Characteristics of randomized controlled trials assessing various Dance-based movement interventions in patients with mild cognitive impairment, Parkinson’s disease, or Alzheimer’s disease.

| Experimental group vs control group | Participants (n) and condition | Dance therapist involved | Blinding | Quality (0-10 score) |  |
| --- | --- | --- | --- | --- | --- |
| Irish set dancing classes vs physiotherapy (Volpe et al., 2013) | 24 PD participants (male/female=13/11; mean age= 63.3±4.9yr; mean disease duration= 8.95±3.05yr; mean Hoehn-Yahr Score= 2.2±0.4; mean MMSE score= 26.4±1.6); | No | Single (assessors) | Moderated  (6) |  |
| Irish set dancing classes vs exercises or usual care (Shanahan et al., 2017) | 41 PD participants (male/female=26/15; mean age= 69±9yr; mean disease duration= 5.75±7yr; mean Hoehn-Yahr Score= 1.625±1); | No | Single (assessors) | Moderated  (6) |  |
| Sardinian folk dance vs usual care (Solla et al., 2019) | 20 PD participants (male/female=13/7; mean age= 67.4±6.1yr; mean disease duration= 4.7±3.7yr); | No | Single (assessors) | Moderated (6) |  |
| Tango, parted vs usual care (Rios Romenets et al., 2015) | 33 PD participants (male/female=19/14; mean age= 63.75±9yr; mean disease duration= 6.6±4.5yr); | No | No | Moderated  (6) |  |
| Tango single vs usual care (Michels et al., 2018) | 13 PD participants (male/female=19/14; mean age= 70.97yr; mean Hoehn-Yahr Score= 2.31±0.67; mean MoCA= 26.13±1.84); | No | No | Moderated  (4) |  |
| Tango vs Tai Chi (Poier et al., 2019) | 29 PD participants (male/female=12/17; mean age= 68.69±9.52yr; disease duration>10 years= 16 participants); | No | No | Moderated  (5) |  |
| Group/Partnered vs group structured strength/flexibility exercise (Hackney et al., 2007) | 19 PD participants (male/female=12/7; mean age= 71.1±2.15yr; mean disease duration= 4.75±1yr; mean Hoehn-Yahr Score= 2.25±0.65; mean Total UPDRS Motor Subscale Score= 29.4±1.25); | No | Single (assessor) | Moderated  (5) |  |
| Partnered vs Nonpartnered tango (Hackney & Earhart, 2010) | 39 PD participants (male/female=28/11; mean age= 69.6±9yr; mean disease duration= 8.7±5yr; mean Hoehn-Yahr Score= 2.25±1.3; mean Total UPDRS Motor Subscale Ⅲ Score= 30.15±8.5); | No | Single (participants) | Moderated  (6) |  |
| Tango vs waltz/foxtrot or no intervention (control) groups (Hackney & Earhart, 2009) | 58 PD participants (male/female=34/14; mean age= 67.17±2.2yr; mean disease duration= 7.33±1.27yr; mean Hoehn-Yahr Score= 2.1±0.17; mean Total UPDRS Motor Subscale Ⅲ Score= 27.3±2.3); | No | Double (participants, assessor) | Moderated  (5) |  |
| Partnered community-based tango vs no intervention control group (Foster et al., 2013) | 52 PD participants (male/female=30/22; mean age= 69.15±8.6yr; mean disease duration= 6.4±5.1yr; mean Total UPDRS Motor Subscale Ⅲ Score= 46.25±10.45); | No | Single (assessor) | High  (7) |  |
| Partnered community-based tango vs no intervention control group (Duncan & Earhart, 2012) | 62 PD participants (male/female=35/27; mean age= 71.33±3.3yr; mean disease duration= 11.15±1.23yr; mean Hoehn-Yahr Score= 2.56±0.2); | No | Single (assessor) | High  (7) |  |
| Partnered community-based tango vs no prescribed exercise control group (Duncan & Earhart, 2014) | 10 PD participants (male/female=8/2; mean age= 67.8±8.8yr; mean disease duration= 8.8±5.7yr; mean Total UPDRS Motor Subscale Ⅲ Score= 46.25±10.45); | No | Single (assessor) | Moderated  (5) |  |
| Turo (mixed Qigong dance) (Lee H. J. et al., 2018) | 20 PD patients (male/female=10/10; mean age= 65.7 ± 6.8yr); | No | Single (assessor) | Moderated  (6) |  |
| Double ballroom and Latin  American dance vs usual care (Hulbert et al., 2017) | 27 PD patients (male/female=12/15; mean age= 72.55± 5yr; mean disease duration= 5.7± 4.05yr; mean Hoehn-Yahr Score= 1.85± 0.9); | No | Single (Statistical analysis) | Moderated  (5) |  |
| Incorporated strategies-based dance (mixed by aerobic, jazz, classical ballet, and tango) vs PD exercise (Hashimoto et al., 2015) | 46 PD patients (male/female=12/34; mean age= 66.77± 8.63yr; mean disease duration= 7± 4.93yr; mean MMSE Score= 28.33± 2); | No | No | Moderated  (6) |  |
| Dance-physiotherapy combined intervention vs conventional physiotherapy (Frisaldi et al., 2021) | 38 PD patients (male/female=12/34; mean age= 66.77± 8.63yr; mean disease duration= 7± 4.93yr; mean MMSE Score= 28.33± 2); | No | Singles (assessor) | Moderated  (6) |  |
| Binary vs quaternary dance rhythm (Moratelli et al., 2021) | 31 PD patients (male/female=22/9; mean age= 69± 8.75yr; mean MoCA Score= 18.4± 5.8; mean QoL score= 54.3± 30.2; mean UPDRS-1 score= 3.23±2; mean UPDRS-2 score= 13.7±8.4); | No | / | Moderated  (5) |  |
| Virtual reality dance exercise vs usual care (Lee N. Y. et al., 2015) | 20 MS patients (male/female=10/10; mean age= 69.25±3.1yr); | No | No | Moderated  (4) |  |

PD= Parkinson's disease; yr= year;

High quality: total score ≥7; moderate quality: total score 4–6; low quality: total score ≤3.
